# Serum cortisol level and survival rate of juvenile *Epinephelus fuscoguttatus* following exposure to different salinities

**DOI:** 10.14202/vetworld.2018.327-331

**Published:** 2018-03-17

**Authors:** Diyana Tahir, M. Shariff, Fadhil Syukri, F. M. Yusoff

**Affiliations:** 1 Laboratory of Marine Biotechnology, Institute of Bioscience, Universiti Putra Malaysia, Malaysia; 2 Department of Veterinary Clinical Studies, Faculty of Veterinary Medicine, Universiti Putra Malaysia, Malaysia; 3 Department of Aquaculture, Faculty of Agriculture, Universiti Putra Malaysia, Malaysia

**Keywords:** cortisol, Epinephelus fuscoguttatus, salinity, serum, stress, survival

## Abstract

**Background and Aim:**

Brown-marbled grouper *Epinephelus fuscoguttatus* is a premium marine food fish with high demand in Asia. In fish, stress due to environmental changes such as fluctuations in the salinity can result in increased cortisol level. Stress in fish increases susceptibility to diseases ultimately resulting in death. Therefore, the aim of this study was to investigate the salinity tolerance of *E. fuscoguttatus* and their survival in lower salinities.

**Materials and Methods:**

In this study, grouper juveniles (92.43±standard error of the mean 0.51 mm) maintained in 31 ppt seawater were transferred into five tanks with seawater diluted to 25, 20, 15, 10, and 5 ppt. The salinity of the control group was not changed and was maintained at 31 ppt. Serum cortisol was measured using ELISA at 0, 30, 60, and 120 min after the fish were transferred to the different concentrations of salinity.

**Results:**

The survival percentage was recorded for 14 days following the transfer and the results revealed that serum cortisol of fish in a high change in salinity (15, 10, and 5 ppt) was significantly higher than the control group immediately after exposure. At the high salinity change, the cortisol levels gradually decrease at 30 min and 60 min, until no difference in cortisol concentration was observed at 120 min. No mortality was observed in fish exposed to low salinity change (25 and 20 ppt) while in higher salinity change (5 ppt), the survival percentage was 50%.

**Conclusion:**

The study revealed that the serum cortisol concentration was high initially and continues to decrease to resting cortisol level at 120 min indicating that cortisol hormone is released following acute stress as a primary response in grouper juveniles.

## Introduction

Brown-marbled groupers (*Epinephelus fuscoguttatus*) belong to the class Actinopterygii, order Perciformes, and family Epinephelidae [1]. This species of grouper is widely distributed in the Indo-pacific region [2] and inhabit lagoon, channels, reef slopes, and coral-rich areas with clear waters. Epinephelidae groupers are the most commercially important groups of tropical marine fish globally, commanding a high price in markets and being heavily targeted in fisheries [3,4], and hence, is categorized as near threatened by the International Union for Conservation of Nature [5]. It is cultured mainly in countries including China, Taiwan, Indonesia, and Malaysia [6] in floating cages with seed production mainly from the wild broodstock [7].

Intensive farming exposes groupers to a variety of stress such as capture and handling, overcrowding, and changes in the water conditions [8]. Change in water salinity affects the physiological processes in the fish [9]. The biochemical processes inside and outside cells are greatly influenced by salinity, and marine teleost actively secretes salt and retain water to maintain osmotic balance in their body [10]. Hormones that become elevated during an event of stress include thyroxine, prolactin, and somatolactin but cortisol acts as the main corticosteroid regulating water balance mechanisms [11]. Cortisol is a steroid hormone that is responsible for various biological activities including gluconeogenesis and immunosuppressive [12]. In teleost, stress responses involve primary, secondary, and tertiary responses [13]. However, cortisol is associated with primary response, and therefore, is an effective way to measure this response and the degree of stress experienced by the fish.

Low survival during culture is attributed to stress and diseases because of poor quality seeds from broodstock [14] making it one of the most difficult fish to culture. The practice of culturing groupers in floating cages also exposes the fish to variability in salinity as a result of tropical weather condition of heavy rainfalls [15] with storm seasons [16] and poor water quality [17]. Therefore, there is a need to produce quality seeds that are stress tolerant. Selective breeding programs by selection of resistant traits can be a reliable method and serve as a long-term solution to control disease problems [18]. Economically important traits such as stress tolerant can be utilized as marker-assisted selective breeding using DNA markers such as microsatellites [19] and single nucleotide polymorphisms [20]. Studies on the salinity affects to the brown-marbled grouper could be used for optimizing culture conditions, and furthermore, stress tolerant fish can be used for selective breeding. The present study investigated the serum cortisol concentration and the survival rate of brown-marbled grouper juveniles following exposure to different salinities.

## Materials and Methods

### Ethical approval

In this investigation, ethical approval was obtained from Institutional Animal Care and Use (IACUC) Universiti Putra, Malaysia.

### Exposure of juveniles to different salinities

**Figure-1:**
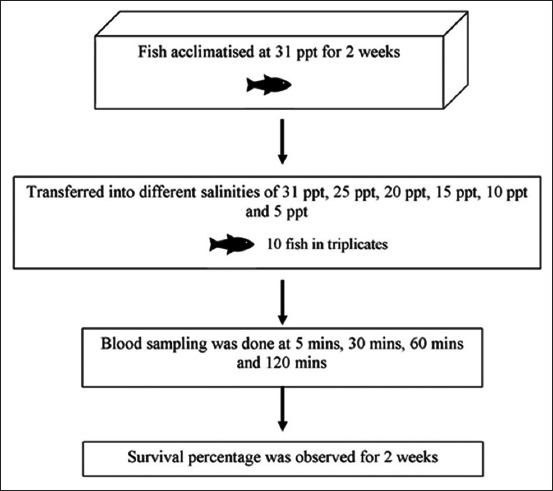
The experimental design for measuring serum cortisol level in *Epinephalus fuscoguttatu*s exposed to different salinities. Two hundred fingerlings were acclimatized in a 500 L tank with 31 ppt salinity for 2 weeks. After acclimatization, 10 fish were introduced into each tank containing 22 L seawater adjusted at different salinities of 31 ppt, 25 ppt, 20 ppt, 15 ppt, 10 ppt and 5 ppt with triplicates. Blood sampling was done after 5, 30, 60 and 120 min. followed by observation for survival percentage for two weeks.

Two hundred brown-marbled grouper juveniles of 92.43±standard deviation 0.51 mm in total length were purchased from a private farm in Sepang, Selangor. Figure-1 shows the experimental design for measurement of serum cortisol level in *E. fuscoguttatus* exposed to different salinities. The fish were acclimatized in a 500 L tank for a period of 2 weeks in salinity of 31 ppt and at water temperature of 27-30°C. For the experiment on exposure of fish to different salinities (31 ppt, 25 ppt, 20 ppt, 15 ppt, 10 ppt, and 5 ppt), triplicate tanks measuring 405×223×253 mm were set up for each salinity concentration. Seasoned tap water was used to dilute seawater to achieve the different salinities. The salinity was measured using 556 MPS Water Quality and Sampling Meter (YSI, USA). Following acclimatization of fish in 31 ppt, 10 fish were introduced into each tank containing 22 L seawater adjusted at different salinity dilutions. The juveniles were fed with commercial sinking marine pellet to satiety 2 times daily. Excess feed left uneaten was siphoned out manually.

### Sampling of blood for cortisol levels

Fish were fasted for 12 h before blood sampling for measuring cortisol levels. The fish were anesthetized with tricaine methanesulfonate (MS-222) with the dosage 100 mg/L. First blood samples were taken 5 min after the fish were transferred to the tanks containing seawater at different salinities, followed by sampling at 30 min, 60 min, and 120 min for each group. Blood was collected through the caudal tail venipuncture. Blood from 3 fish per group per time was pooled and stored in a 1.5 ml microcentrifuge tube and left to stand for 2 h before centrifuged at 9000 rpm for 15 min at room temperature. The serum was collected and then stored at −20°C for serum cortisol ELISA analysis. Serum cortisol level was determined using Fish Cortisol ELISA kit (CusaBio, USA). Briefly, the sample containing the cortisol antibody was added to each antigen pre-coated wells in duplicates. The plate was then incubated for 40 min at 37°C. The wells were washed 3 times to remove unbounded antibodies and then horseradish peroxidase (HRP)-conjugate was added and the plate was incubated for 30 min at 37°C. After another washing, tetramethylbenzidine substrate was added for detection of HRP activity and the plate was incubated for 20 min at 37°C. A stop solution was added and mixed well by gentle shaking. The cortisol level was determined by reading the optical density at 450 nm with reference reading to 540 nm using Infinite M200Pro Spectrophotometer (Tecan, Switzerland). A standard curve was calculated using eight standards, and the serum concentration was extrapolated from this curve with r^2^=0.9999. One-way ANOVA multiple comparisons were used to analyzed the data. Statistical difference was analyzed at p<0.05.

The fish were then maintained in the aquaria to determine the survival rate for 14 days. Partial water change was done for all tanks every 2 days while maintaining the original salinity dilutions for each group.

## Results and Discussion

### Cortisol serum level

**Figure-2:**
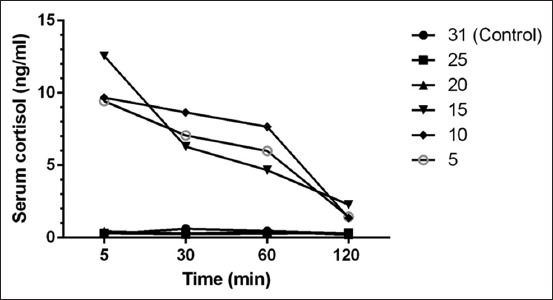
Time-course changes of mean serum cortisol (ng/ml) of *E. fuscoguttatu*s under different salinities.

Figure-2 shows that the serum cortisol level at 5 min sampling of fish exposed to higher change of salinities of 15 ppt, 10 ppt, and 5 ppt was 12.5612 ng/ml, 9.6850 ng/ml, and 9.4351 ng/ml, respectively, whereas the serum cortisol concentration in low change in salinities (25 ppt and 20 ppt) ranged from 0.2972 ng/ml to 0.4379 ng/ml. There was a significant difference of the mean cortisol concentration of these dilutions compared to the control level. Following this, the serum cortisol concentration for the high change of salinity decreased with time ranging from 12.56120 ng/ml to 1.36822 ng/ml. There was no significant difference (p<0.05) between the mean serum cortisol level in fish in lower change of salinity; 25 ppt (0.2803 ng/ml±standard error of the mean [SEM] 0.01685) and 20 ppt (0.338 ng/ml±SEM 0.05588) compared to the control fish at 31 ppt (0.4115 ng/ml±SEM 0.08135). Cortisol is elevated after the fish transfer to tanks with higher salinity change as cortisol is a hormone implicated in osmoregulatory processes [12] with an important role in adaptation to hypertonic or hypotonic environments [21]. Cortisol hormone main target tissue is the gill chloride cell and activates Na^+^–K^+^ ATPase activity [22], therefore, is an important hormone for seawater and ion uptake [23]. In the present study, observation of the fish after direct transfer showed signs of agitation for 5-10 min shown by erratic swimming which was similar to findings by Árnason *et al*. [24], whereby abnormal behaviors such as swimming upside down were reported at dilutions of 15 ppt, 10 ppt, and 5 ppt. In addition, the present study showed darker body color after transfer which then returns to the original state after 30 min.

In teleost, previous studies have reported the cortisol level ranging from 5 ng/ml to 30 ng/ml [13,25]. For example, Tintos and colleague investigate the serum cortisol concentration in four species of fish, namely, the rainbow trout (*Oncorhynchus mykiss*), gilthead sea bream (*Sparus aurata)*, Senegales sole (*Solea senegalensis)*, and sea bass (*Dicentrarchus labrax)* in control and stressed fish. The fish were stressed with short-term exposure to the air, increased stocking density and chasing. Their findings revealed the basal or resting cortisol concentration ranged from 5.65±0.21 to 26.3±1.19 ng/ml while stressed fish cortisol level ranged from 24.2±0.84 to 114.6±13.46 ng/ml. Although the serum cortisol level in the present study was comparatively low in contrast to the range reported in above-mentioned studies, the findings are similar to several studies on cortisol in *Epinephelus* sp. [26]. The study by Raihan *et al*. stressed hybrid tiger grouper (*E. fuscoguttatus*) x giant grouper (*Epinephelus lanceolatus*) with 7 salinity treatments; 5, 10, 15, 20, 25, and 35 ppt diluted from 30 ppt (control). In their study, significantly higher cortisol levels were detected in fish in high salinity change (5 and 15 ppt) and low salinity change (35 ppt) which ranged from 4.5856 ng/ml to 20.4813 ng/ml. Thus, basal and stressed cortisol differs between species, and interspecies are affected by factors including temperature, gender, sexual maturity, and genetics [27].

No significant difference was observed in serum cortisol level among fish in all the salinity changes at 120 min post-challenge. This is in agreement with the findings of Tsui *et al*. [28], which measured 5 test dilutions of 14, 19, 29, 34 ppt, and 24 ppt (control), and performed blood sampling at 30 min interval from 0 to 240 min. The concentration of serum cortisol level peaked 10 min after exposure, and significantly increased cortisol concentrations were observed in lower salinity changes of 29 ppt (30.5392 ng/ml) and 34 ppt (35.3311 ng/ml) compared to the control value of 24 ppt (5.9106 ng/ml). No significant difference in the cortisol level was observed in fish in all test solutions after 120 min which is in agreement with our study.

**Figure-3:**
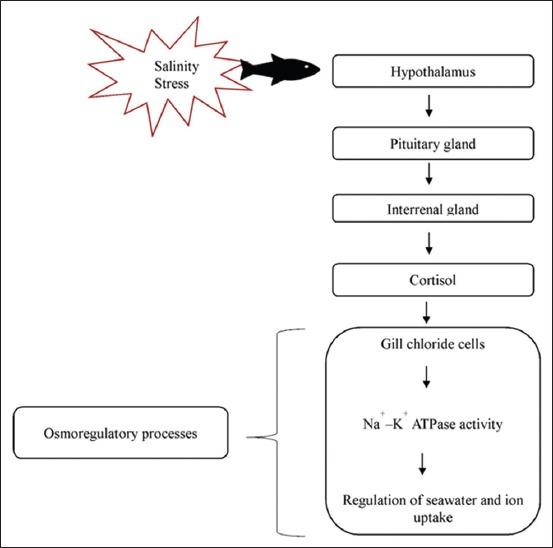
The experimental design for measurement of serum cortisol level in *Epinephelus fuscoguttatus* exposed to different salinities. 200 fingerlings were acclimatized in a 500 L tank with 31 ppt salinity for 2 weeks. After acclimatization, 10 fish were introduced into each tank containing 22 L seawater adjusted at different salinities of 31 ppt, 25 ppt, 20 ppt, 15 ppt, 10 ppt, and 5 ppt with triplicates. Blood sampling was done after 5, 30, 60, and 120 min followed by observation for survival percentage for 2 weeks.

Both *Epinephelus* sp. and tilapia are euryhaline, which can tolerate freshwater and marine environment. Thus, when results of the present study were compared to the report on freshwater tilapia by Ron *et al*. [29], the peak elevation of cortisol was observed within 4 min post-challenge, and then remained elevated for a longer duration of 2-3 days before returning to basal levels. In another study on *Cyprinus carpio* [30], the serum cortisol level peaked was measured at 20 min after cold shock. All of the above studies agree that when fish are exposed to stress, it first shows an acute response which then subsides after a few hours [31]. As shown in Figure-3, the endocrine response in fish is initiated by recognition of stress by the central nervous system followed by the release of corticosteroids [32]. The release of cortisol is controlled by a negative feedback mechanism of the hypothalamic-pituitary-interrenal (HPI) axis equivalent to hypothalamic-pituitary-adrenal in humans and mammals. This acute, primary response is important as the secondary response takes over to produce changes in the metabolism, cellular, osmoregulatory, hematology, and immune function to achieve hemostasis [33]. When the perceived stressors are reduced and HPI axis is no longer stimulated, a delayed negative feedback mechanism takes place [34] that is responsive to glucocorticoid levels. Consequently, the concentration of cortisol will be reduced following adaptation to the changes. However, during chronic stress, plasma cortisol falls back to the resting levels, even though the fish may still be responding to the stressor [35].

### Survival rate following transfer

**Figure-4:**
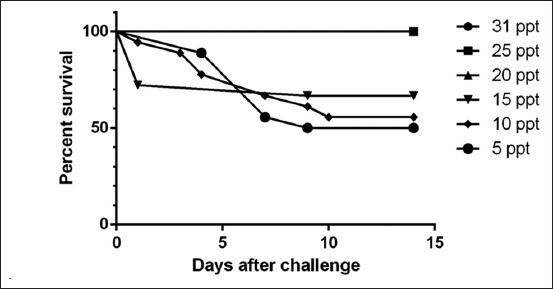
Survival percentage of *E. fuscoguttatu*s 14 days post challenged with different salinities.

In the present study, no mortality was observed in fish from the groups exposed to lower change of salinities of 20 ppt, and 25 ppt, and control 31 ppt (Figure-4) while in higher change of salinities of 15 ppt, 10 ppt, and 5 ppt the survival percentage was 70%, 60%, and 50%, respectively. The results showed that groupers can survive in lower dilutions of salinity.

Groupers have been reported as a strong osmoregulator and can tolerate various salinity levels [28]. In the present study, a direct transfer into tanks with a lower change of salinities did not cause mortalities in the 1^st^ day. This finding is similar to another study on Atlantic cod whereby direct transfer to higher change of salinity of 7 ppt from 26 to 28 ppt did not cause mortalities or showed indication of stress [36]. Fish such as salmons, Atlantic cods, and groupers, which are euryhaline species, are able to tolerate a wider range of salinities while a much narrow range of tolerance is adapted in stenohaline fishes [10]. According to Stickney [37], the ability of body fluid to tolerate changes of osmolality and ion concentrations will affect the survival rate of the fish. In addition, juveniles in their natural habitat are found in shallow waters of estuaries over sand, mud, and gravel, and among mangroves [2]. These areas of estuarine salinity range from 10 to 35 ppt [38]. Juveniles are able to tolerate low salinities and direct transfer from seawater without mortalities although they show signs of stress [28]. In aquaculture setting, adults and juveniles in indoor tanks are raised at salinity ranging from 27 ppt to 31 ppt in a controlled environment as opposed to in the natural habitat which ranges from 10 to 35 ppt.

## Conclusion

Juveniles brown-marbled grouper is tolerant and able to survive in a range of lower change of salinities (25 and 20 ppt) in salinity compared to the control maintained at 31 ppt without a significant increase in the serum cortisol level. Higher salinity changes of 15, 10, and 5 ppt showed an increase in the serum cortisol levels, indicating stress, and increase in the mortality rate.

## Authors’ Contributions

DT contributed in designing and running the experiment, maintaining the fish and preparing the manuscript. MS, FS, and FMY contributed in correcting the manuscript. All authors read and approved the final manuscript.
